# PM_2.5_ impairs macrophage functions to exacerbate pneumococcus-induced pulmonary pathogenesis

**DOI:** 10.1186/s12989-020-00362-2

**Published:** 2020-08-04

**Authors:** Yu-Wen Chen, Mei-Zi Huang, Chyi-Liang Chen, Chieh-Ying Kuo, Chia-Yu Yang, Chuan Chiang-Ni, Yi-Ywan M. Chen, Chia-Ming Hsieh, Hui-Yu Wu, Ming-Ling Kuo, Cheng-Hsun Chiu, Chih-Ho Lai

**Affiliations:** 1 Graduate Institute of Biomedical Sciences, Department of Microbiology and Immunology, College of Medicine, Chang Gung University, Taoyuan, Taiwan; 2 Department of Pediatrics, Molecular Infectious Disease Research Center, Chang Gung Memorial Hospital, Linkou, Taiwan; 3 Molecular Medicine Research Center, Chang Gung University, Taoyuan, Taiwan; 4 Department of Otolaryngology-Head and Neck Surgery, Chang Gung Memorial Hospital, Taoyuan, Taiwan; 5 Division of Allergy, Asthma, and Rheumatology, Department of Pediatrics, Chang Gung Memorial Hospital, Linkou, Taiwan; 6 Department of Microbiology, School of Medicine, China Medical University, Taichung, Taiwan; 7 Department of Nursing, Asia University, Taichung, Taiwan

**Keywords:** PM_2.5_, Macrophage, Pneumococcus, Pulmonary inflammation

## Abstract

**Background:**

Pneumococcus is one of the most common human airway pathogens that causes life-threatening infections. Ambient fine particulate matter (PM) with aerodynamic diameter ≤ 2.5 μm (PM_2.5_) is known to significantly contribute to respiratory diseases. PM_2.5_-induced airway inflammation may decrease innate immune defenses against bacterial infection. However, there is currently limited information available regarding the effect of PM_2.5_ exposure on molecular interactions between pneumococcus and macrophages.

**Results:**

PM_2.5_ exposure hampered macrophage functions, including phagocytosis and proinflammatory cytokine production, in response to pneumococcal infection. In a PM_2.5_-exposed pneumococcus-infected mouse model, PM_2.5_ subverted the pulmonary immune response and caused leukocyte infiltration. Further, PM_2.5_ exposure suppressed the levels of CXCL10 and its receptor, CXCR3, by inhibiting the PI3K/Akt and MAPK pathways.

**Conclusions:**

The effect of PM_2.5_ exposure on macrophage activity enhances pneumococcal infectivity and aggravates pulmonary pathogenesis.

## Background

Particulate matter (PM) is a complex mixture of solid and liquid particles released into the environment during coal, petroleum, and fossil fuel combustion [1]. PM with aerodynamic diameter ≤ 2.5 μm (PM_2.5_) is known to significantly contribute to airway inflammation [2–4]. Epidemiological studies have demonstrated that anthropogenic PM_2.5_ exposure was associated with the exacerbation of respiratory diseases, all-cause mortality, and cardiopulmonary mortality [5–7]. Low PM_2.5_ exposure levels also pose certain public health risks [8].

Pneumococcus, a Gram-positive coccus, is the most common cause of global pneumonia mortality [9]. Alveolar macrophages are mainly responsible for the pulmonary defense against pneumococcal infection [10]. Activated macrophages release inflammatory mediators, including IL-1α/β, IL-6, TNF-α, IFN-α/β, CXCL10, MCP-1, and nitric oxide (NO), recruiting nearby immune cells against pneumococcal infection [11]. Alveolar macrophage depletion increased mortality and lung bacterial burden [12], indicating that macrophages play a crucial role in protective anti-inflammation in pneumococcal pneumonia.

Mounting evidence shows that exposure to ambient pollution particles impairs pulmonary functions and favors infectious diseases [13–15]. For instance, concentrated ambient particles enhance pneumococcus binding to macrophages, but decrease its internalization [16]. Coal fly ash (CFA) impairs the antimicrobial peptide (AMP) function that increases *Pseudomonas aeruginosa* growth [17]. Recently, CFA was demonstrated to adsorb to and complex with AMP, decreasing its antimicrobial activity [18]. These lines of evidence indicate that ambient particles influence the airway immune defense, increasing bacterial infection susceptibility.

Ambient particles have been found in airway macrophages in the septum of healthy individuals [19], and their impact on human immune responses have been reported [15, 18, 20]. However, the pathophysiological relevance of PM_2.5_ exposure in the respiratory tract, particularly with respect to macrophages against bacterial infection, remains unclear. In this study, we aimed to investigate the effects of long-term PM_2.5_ exposure on macrophage activation against pneumococcal infection. We established a PM_2.5_-loaded murine model that was pneumococcus-infected to investigate macrophage functions and pulmonary pathogenesis. Further, molecular studies were performed to determine the effect of PM_2.5_ on signaling pathways and whether it acts as an immune suppressor in response to bacterial challenge, leading to the exacerbation of pneumococcus-induced lung pathogenesis.

## Methods

### Antibodies and reagents

Antibodies against PI3K, p-Akt, t-Akt, p-p38, t-p38, p-JNK, t-JNK, p-Erk, t-Erk, and p-p65 were purchased from Cell Signaling Technology (Beverly, MA). Antibody specific to β-actin was obtained from Santa Cruz Biotechnology (Santa Cruz, CA). Antibodies against inducible nitric oxide synthase (iNOS) and high mobility group box 1 (HMGB1) were purchased from Abcam (Cambridge, UK). Antibodies specific to t-p65 and CXCR3 were purchased from GeneTex (Irvine, CA) and Novus Biologicals (Centennial, CO), respectively. Inhibitors specific to Erk (PD98059), p38 (SB203580), JNK (SP600125), and NF-κB (JSH-23) were purchased from Sigma-Aldrich (St Louis, MO).

### Cell and bacterial culture

Macrophage cell line RAW264.7 (ATCC TIB-71) cells were cultured in Dulbecco’s Modified Eagle Medium (DMEM) (Invitrogen, Carlsbad, CA) containing 10% complement-inactivated fetal bovine serum (HyClone, Logan, UT) and incubated at 37 °C in a humid atmosphere containing 5% CO_2_ [21]. *Streptococcus pneumoniae* strain TIGR4 (virulent serotype 4, ATCC BAA-334) was cultured on blood agar plates (Becton Dickinson, Sparks, MD) and incubated at 37 °C under 5% CO_2_ [22]. The bacteria were refreshed for 3 h in Todd Hewitt Broth (Becton Dickinson) to reach the logarithmic phase and were then used in the following infection experiments.

### Characterization of particulate matter

Particulate matter less than 2.5 μm diameter (PM_2.5_) (RM8785) was purchased from National Institute of Standards and Technology (MD, USA) [23]. The particulate matter on filter media was fixed and coated with gold by an ion sputter (E-1010, Hitachi, Japan). The particle size was verified by field-emission scanning electron microscope (FE-SEM) (JSM 7500F, JEOL, Japan).

### Cell viability assay

RAW264.7 cells (1 × 10^5^) were seeded in 96-well plates and treated with low (5 μg/ml) or high (20 μg/ml) doses of PM_2.5_ for 24 h. Cells were incubated with 0.5 mg/ml 3-(4,5-dimethylthiazol-2-yl)-2,5-diphenyltetrazolium bromide (MTT) solution for 2 h. Formazan crystals were dissolved in isopropanol, and the absorbance at 570 nm was determined by a spectrophotometer (Bio-Rad, Hercules, CA) [24]. The cell viability was expressed as a percentage compared to PM_2.5_-untreated group.

### Phagocytosis assay

The Phagocytosis Assay Kit (IgG FITC) (Cayman Chemical, Ann Arbor, MI) was employed to analyze whether PM_2.5_ affects the phagocytic activity of macrophages [25]. RAW264.7 cells (2 × 10^6^) were treated with low (5 μg/ml) or high (20 μg/ml) doses of PM_2.5_ for 24 h. Latex beads coated with fluorescent-labeled rabbit IgG were incubated with cells at 37 °C for 3 h. The cells were fixed with 4% paraformaldehyde followed by staining with Hoechst 33342 (AAT Bioquest, Sunnyvale, CA). The signals of fluorescein isothiocyanate (FITC) and Hoechst 33342 were analyzed under a Laser Scanning Confocal Microscope (LSM780, Carl Zeiss, Germany).

### Bacterial internalization assay

A gentamicin protection assay was used to analyze the bacterial internalization by macrophages [26]. Briefly, RAW264.7 cells were treated with low (5 μg/ml) or high (20 μg/ml) doses of PM_2.5_ for 24 h prior to infection with pneumococcus (MOI = 10) for 6 h. The infected cells were treated with gentamicin (100 μg/ml) for 1.5 h to kill extracellular bacteria. The cells were lysed with sterilized water and cell lysates were seeded on blood agar plates by serial dilution. Visible colony-forming units (CFU) were calculated to determine the bacterial internalization activity.

### Western blot assay

RAW264.7 cells (2 × 10^6^) were untreated or treated with PM_2.5_ (20 μg/ml) for 24 h followed by infection with pneumococcus (MOI = 10) for an additional 6 h. Cells were washed and lysed with 100 μl RIPA containing protease and phosphatase inhibitors (Roche, Indianapolis, IN), and then subjected to western blot assay. The samples were resolved by 12% SDS-PAGE and transferred onto polyvinylidene difluoride membranes (Millipore, Billerica, MA). The membranes were blocked by 5% skim milk and incubated with the primary antibodies followed by incubation with horseradish peroxidase (HRP)-conjugated secondary antibodies (Millipore). The proteins of interests were detected using ECL Western Blotting Detection Reagent (BIOMAN, Taipei, Taiwan) and analyzed by Azure C400 (Azure Biosystems, Dublin, CA) and AzureSpot Analysis Software (Azure Biosystems) [27]. To determine the intensities of western blot bands, Un-Scan-It v6.1 software (Orem, UT, USA) was used. Identical areas surrounding each band were cropped, and protein expression levels were converted into pixel densities. Each area value was normalized to the β-actin density in the same lane on the gel, and then divided by the normalized density in the mock-control. Fold change represents protein expression level relative to the mock-control.

### Analysis of nitric oxide production

RAW264.7 cells (1 × 10^5^) were untreated or treated with 20 μg/ml PM_2.5_. After incubation for 24 h, the cells were infected with pneumococcus (MOI = 10) for 6 h. The culture medium was collected and the nitric oxide production was assessed by using Griess reagent (Sigma-Aldrich) [28].

### Quantitative real-time reverse transcription-PCR (qRT-PCR)

To explore the mRNA levels of iNOS, CD80, CD86, CD163, CD206, and F4/80 in macrophages, we performed qRT-PCR analysis in this study. The oligonucleotide primers for qRT-PCR quantification are shown in Table S1. The mRNA levels were analyzed by qRT-PCR using SYBR Green I Master Mix and a model 7900 Sequence Detector System. The program was pre-incubated at 50 °C for 2 min and 95 °C for 10 min; PCR was performed with 35 cycles of 95 °C for 10 s and 60 °C for 1 min. The data for each gene quantity was determined by relative calculation using the 2^−ΔΔCt^ method. The method was used to calculate fold changes in each treatment group.

### Determination of cytokine production

RAW264.7 cells were untreated or treated with 20 μg/ml PM_2.5_ for 24 h, the cells were uninfected or infected with pneumococcus (MOI = 10) for 6 h. The supernatant was collected from cell culture, and the expression levels of sHMGB1, IL-1α, IL-1β, TNF-α, CXCL9, CXCL10, and CXCL11 were analyzed using sandwich enzyme-linked immunosorbent assay (ELISA, R&D Systems, Minneapolis, MN) [29].

### Animal study

Male BALB/c mice (aged 6 weeks) were obtained from the National Laboratory Animal Center (Taipei, Taiwan). The mouse experiments were performed in accordance with the Animal Care and Use Guidelines for Chang Gung University under a protocol approved by the Institutional Animal Care Use Committee (IACUC Approval No.: CGU16–019). Mice were divided into four groups for the treatments with PBS (mock), PM_2.5_, pneumococcus, and PM_2.5_ + pneumococcus (10 mice per group; 6 mice for harvesting bronchoalveolar lavage fluid (BALF) and 4 mice for histopathological examination). PM_2.5_ was administered by intratracheal (i.t.) instillation twice per week for 3 weeks (total amount of PM_2.5_ = 200 μg). Amongst, two mice in PM_2.5_ + pneumococcus group died during PM_2.5_ exposure period and were excluded in the following studies. Mice were placed in the chambers, allowed to rest for 4 days, and then infected with pneumococcus by intranasal (i.n.) injection (1 × 10^8^ CFU/10 μl). After infection for 48 h, the mice were euthanized, and the BALF and lungs were isolated as described previously [21]. Bacterial survival in BALF was analyzed. In each group, 6 mice were used to prepare BALF for cell enumeration and differentiation, while the lung tissues of 4 mice were investigated using H&E and IHC staining. qRT-PCR assay was employed to assess the copy number of pneumococcus genomic DNA in BALF. The oligonucleotide primers used to analyze pneumococcus TIGR 4 were as follows: forward, 5′-GGG GAA GTA TTT TCA GAG TCG-3′; and reverse, 5′-AAT CAC CAA CTA ACC ATC CAA TAG-3′ [30]. The inflammatory cells in BALF were distinguished using Wright–Giemsa stain.

### Cytokine array

BALF prepared from each mouse in the same group were pooled into one sample and analyzed by Proteome Profiler Array (R&D Systems). Images were captured using an Azure C400 (Azure Biosystems, CA). The quantifications of each dot were measured by Image J, and the fold changes were calculated by Log_2_. The expression levels of cytokines were expressed as the average signal intensity of duplicate spots subtracted from signal background and normalized to total protein concentration.

### Histopathological analysis

Lung tissues isolated from mice were prepared for hematoxylin-eosin (H&E) or immunohistochemistry (IHC) staining as described previously [31]. The lung sections were stained with antibodies against CXCR3, IL-1β, and F4/80, respectively, followed by incubation with HRP-conjugated secondary antibodies and developed with an ABC kit (Vector Laboratories, Burlingame, CA). The stained tissues were observed and evaluated by using a microscope (AXIO IMAGER M2, Carl Zeiss, Germany).

### Statistical analysis

Statistical analysis was performed using the SPSS program (version 18.0 for windows, SPSS Inc., Chicago, IL), and all data are shown as mean ± standard deviation (SD). Statistical significance was determined by Student’s *t*-test for two groups and one-way ANOVA with Tukey post-hoc test for more than two groups. *P-*value of less than 0.05 was considered statistically significant.

## Results

### PM_2.5_ impairs phagocytosis of pneumococcus by macrophages

**Fig. 1:**
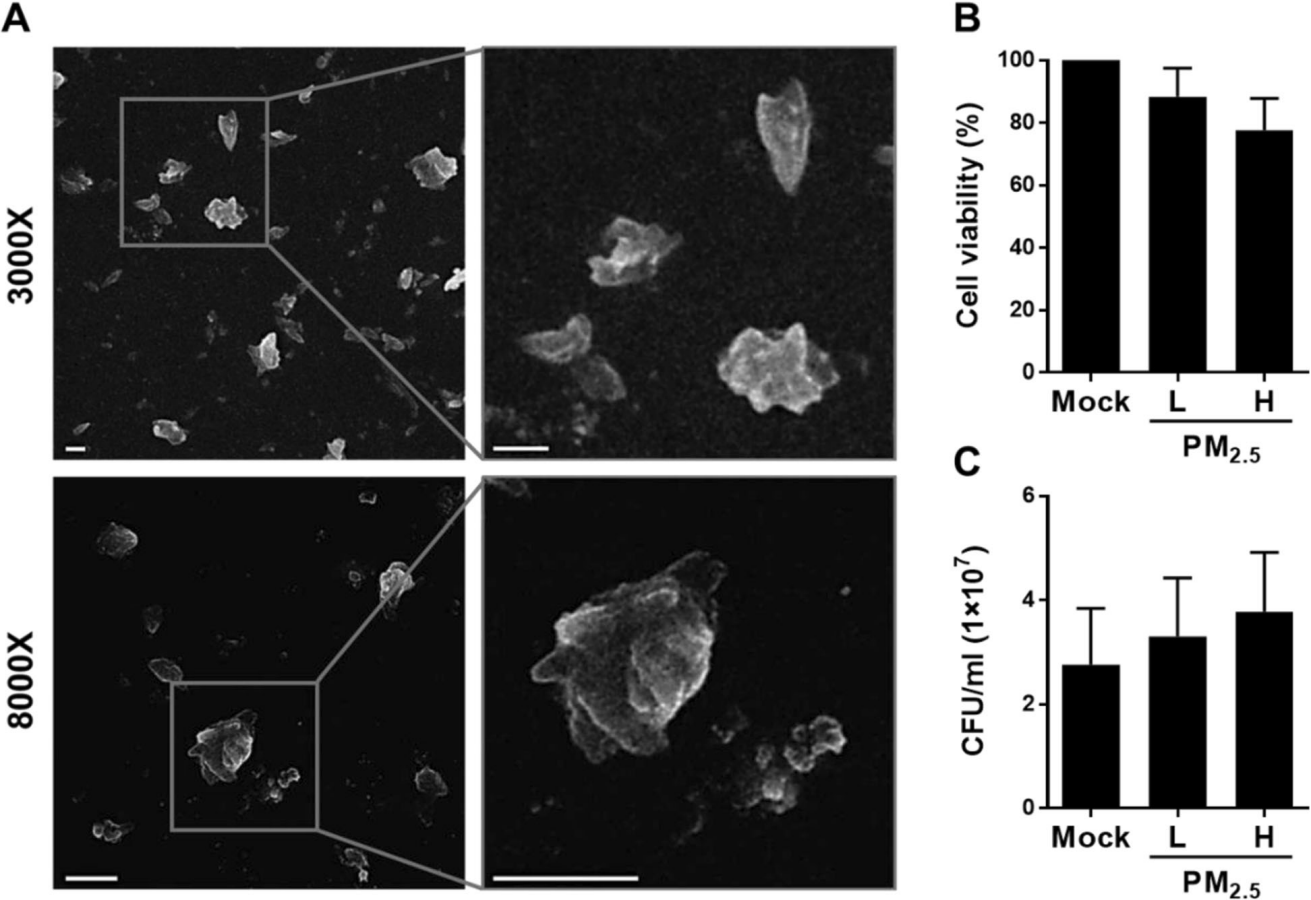
PM_2.5_ does not affect macrophage viability and pneumococcus survival. **a** Transmission electron micrograph of the particulate matter. The magnified images of cropped areas are shown in the right panels. Original magnification: 3000× (upper panel) and 8000× (lower panel). Scale bars, 1 μm. **b** Cell viability of RAW264.7 cells exposed to low (5 μg/ml) or high (20 μg/ml) doses of PM_2.5_ for 24 h, analyzed with MTT assay. **c** Pneumococcus incubated with low or high PM_2.5_ doses for 6 h. Bacteria grown on the blood agar plates were counted and expressed as colony-forming units (CFU). Results are presented as mean ± standard deviation from triplicate independent experiments

We first characterized particulate matter size using FE-SEM. The size distribution was dominated by particles smaller than 2.5 μm (referred to as PM_2.5_) (Fig. 1a). RAW264.7 cells were then treated with 20 μg/ml PM_2.5_ for 24 h, and light microscopy showed that the particulate matter was deposited and likely embedded in the cells (Additional file 1: Fig. S1). We further investigated the influence of PM_2.5_ exposure on macrophage viability by treating RAW264.7 cells with low (5 μg/ml) or high (20 μg/ml) PM_2.5_ doses for 24 h. Cell viability was minimally affected by both doses (Fig. 1b). Additionally, PM_2.5_ exposure only marginally influenced pneumococcal survival (Fig. 1c). Therefore, 5 and 20 μg/ml of PM_2.5_ were employed for subsequent experiments.

**Fig. 2:**
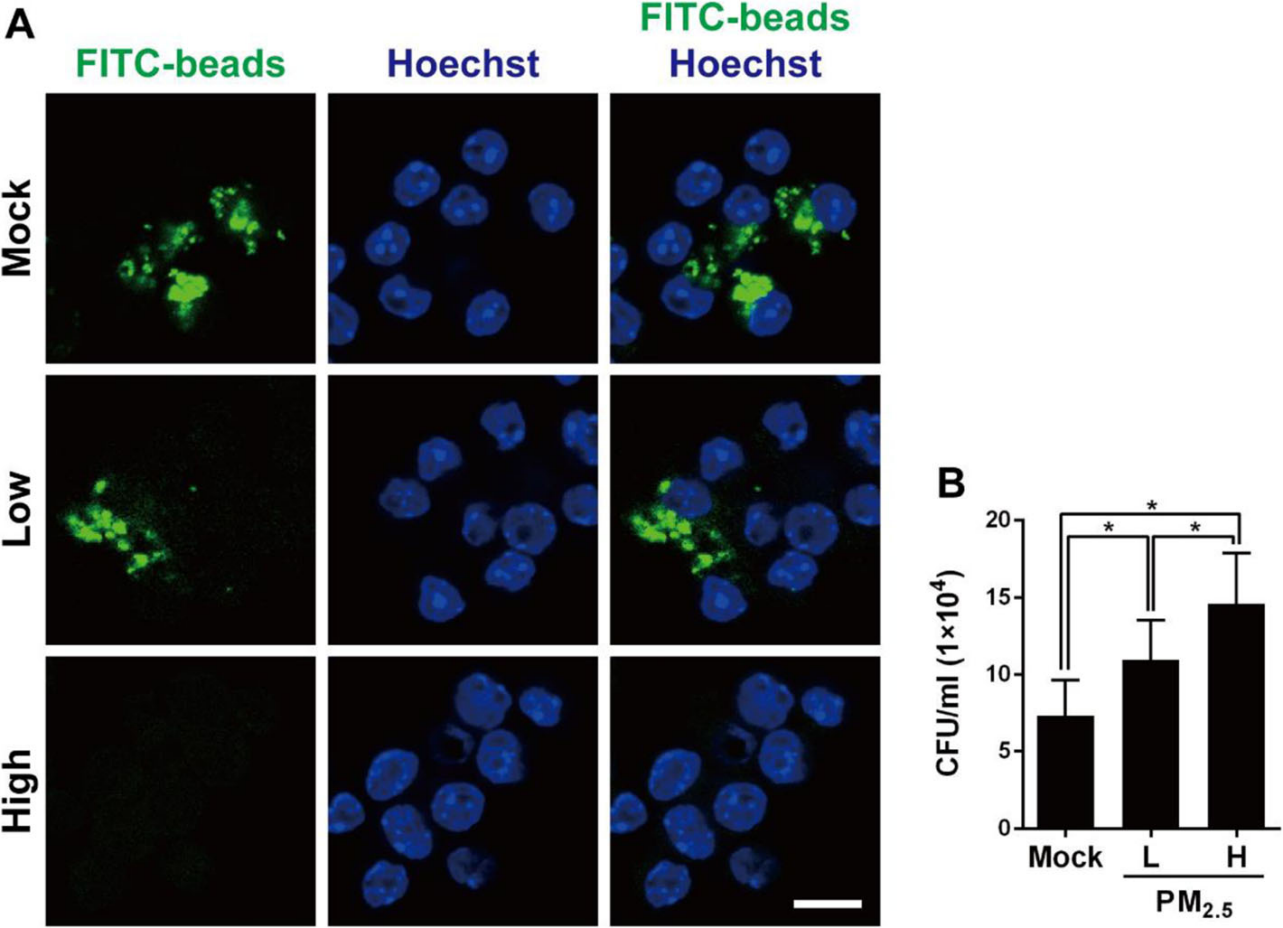
PM_2.5_ suppresses macrophage phagocytosis in response to pneumococcal infection. **a** RAW264.7 cells were exposed to low (5 μg/ml) or high (20 μg/ml) doses of PM_2.5_ for 24 h, and then incubated with latex fluorescent beads for 3 h. The nuclei were stained with Hoechst 33342 and the image was analyzed by confocal microscopy. Scale bar, 10 μm. **b** RAW264.7 cells were exposed to low or high doses of PM_2.5_ for 24 h before pneumococcal infection for 6 h (MOI = 10). Intracellular pneumococcal survival was determined using gentamicin protection assays and expressed as viable CFU. Results are represented mean ± standard deviation from triplicate independent experiments and *P*-value was determined by using one-way ANOVA followed by a post-hoc test (*, *P* < 0.05)

To examine whether PM_2.5_ exposure affects macrophage phagocytic activity, we analyzed phagocytosis using antibody-coated latex beads and fluorescence. Low PM_2.5_ dose (5 μg/ml) slightly reduced macrophage internalization of the latex beads (Fig. 2a). In contrast, high PM_2.5_ dose (20 μg/ml) treatment dramatically reduced latex bead internalization, indicating that PM_2.5_ hinders macrophage phagocytic activity. We further determined bacterial internalization by macrophages using the gentamicin protection assay. As shown in Fig. 2b, the total bacterial survival was significantly increased in RAW264.7 cells treated with both low and high doses of PM_2.5_. These results indicate that PM_2.5_ exposure impairs macrophage phagocytic activity and may reduce pneumococcal clearance.

### PM_2.5_ inhibits macrophage pneumococcus-induced inflammatory mediators

**Fig. 3:**
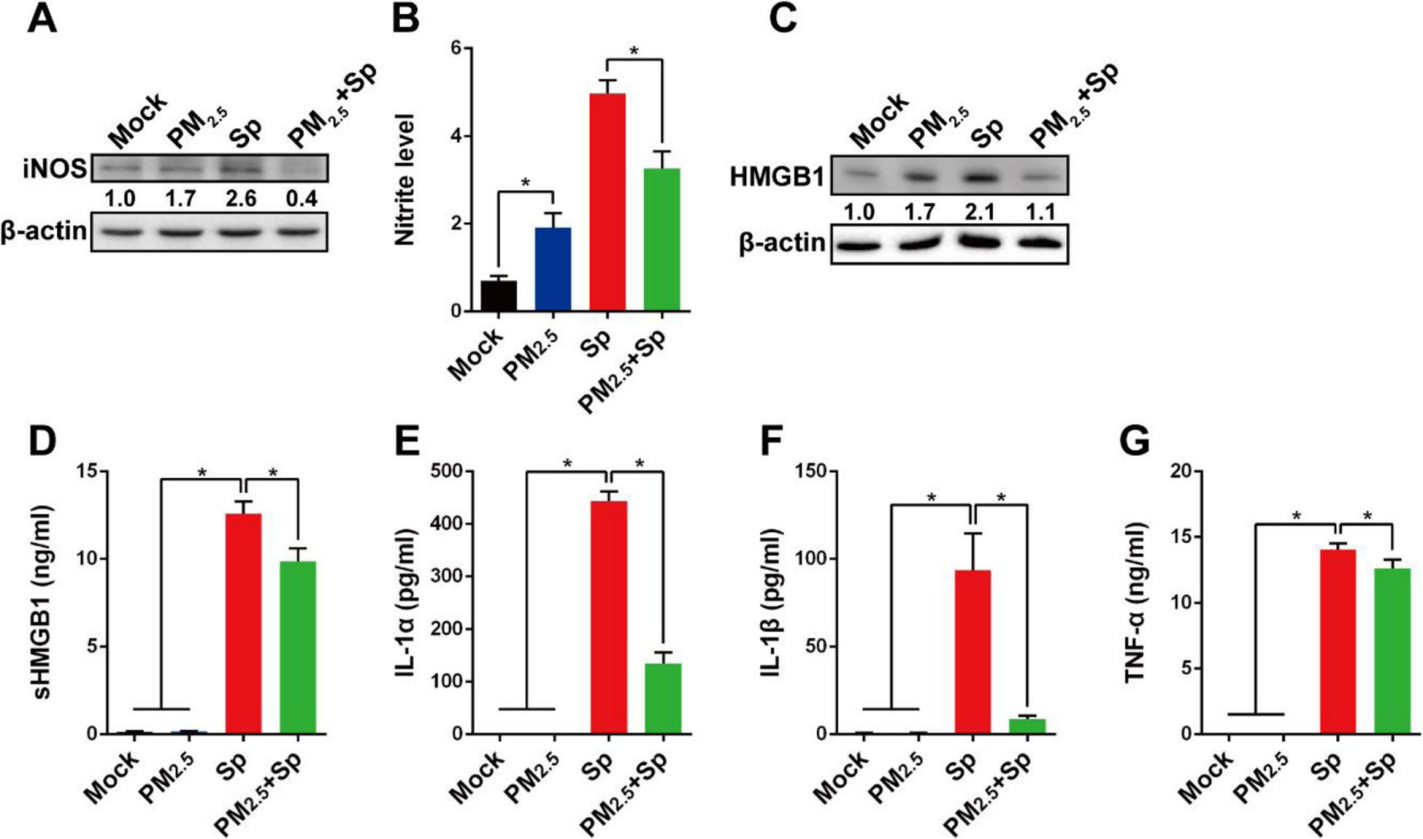
PM_2.5_ dampens pneumococcus-induced inflammatory mediators in macrophages. RAW264.7 cells were unexposed or exposed to 20 μg/ml of PM_2.5_ for 24 h, followed by pneumococcus challenge for 6 h. **a** iNOS expression levels were determined using western blotting, and (**b**) the culture supernatant nitrite concentrations were determined using Griess reagent and normalized to cell viability. **c** HMGB1 expression levels were analyzed using western blotting. Relative protein expression levels were normalized to those in the mock-treated group and are indicated under each band. **d** Secreted HMGB1 (sHMGB1), (**e**) IL-1α, (**f**) IL-1β, and (**g**) TNF-α production in culture supernatant were determined using ELISA. The values were means ± standard deviations from triplicate independent experiments. Statistical significance was evaluated using Student’s *t*-test or one-way ANOVA followed by a post-hoc test (*, *P* < 0.05)

Nitric oxide (NO) generated by inducible nitric oxide synthase (iNOS) in macrophages is known to kill microorganisms [32]. Therefore, the effects of PM_2.5_ on NO production and pneumococcal clearance by macrophages were investigated. Our results showed that iNOS expression was higher in both PM_2.5_-exposed and pneumococcus-infected cells than in untreated mock cells (Fig. 3a). In contrast, pneumococcus-induced iNOS expression was markedly reduced by PM_2.5_-treatment. In parallel, nitrite levels were increased in PM_2.5_-exposed or pneumococcus-infected cells, but significantly reduced in PM_2.5_-treated macrophages that were challenged with pneumococcus (Fig. 3b). These results indicate that PM_2.5_ inhibits pneumococcus-induced iNOS protein expression and reduces nitric oxide production, thereby attenuating macrophage bactericidal activity.

To further investigate the effect of PM_2.5_ on the inflammatory response, we measured the production of several proinflammatory cytokines. HMGB1 exerts proinflammatory activities when released from macrophages [33]. Pneumococcus-induced HMGB1 expression in cells was significantly decreased by PM_2.5_ exposure (Fig. 3c). The same trend was also observed for other macrophage-produced proinflammatory cytokines, including sHMGB1, IL-1α, IL-1β, and TNF-α, which were inhibited by the co-treatment of PM_2.5_ and pneumococcus (Fig. 3d-g). These results demonstrate that PM_2.5_ impedes pneumococcus-induced proinflammatory cytokine production and may attenuate the immune response to bacterial infection.

### Long-term PM_2.5_ exposure subverts pneumococcal clearance in lungs and exacerbates pulmonary pathogenesis

**Fig. 4:**
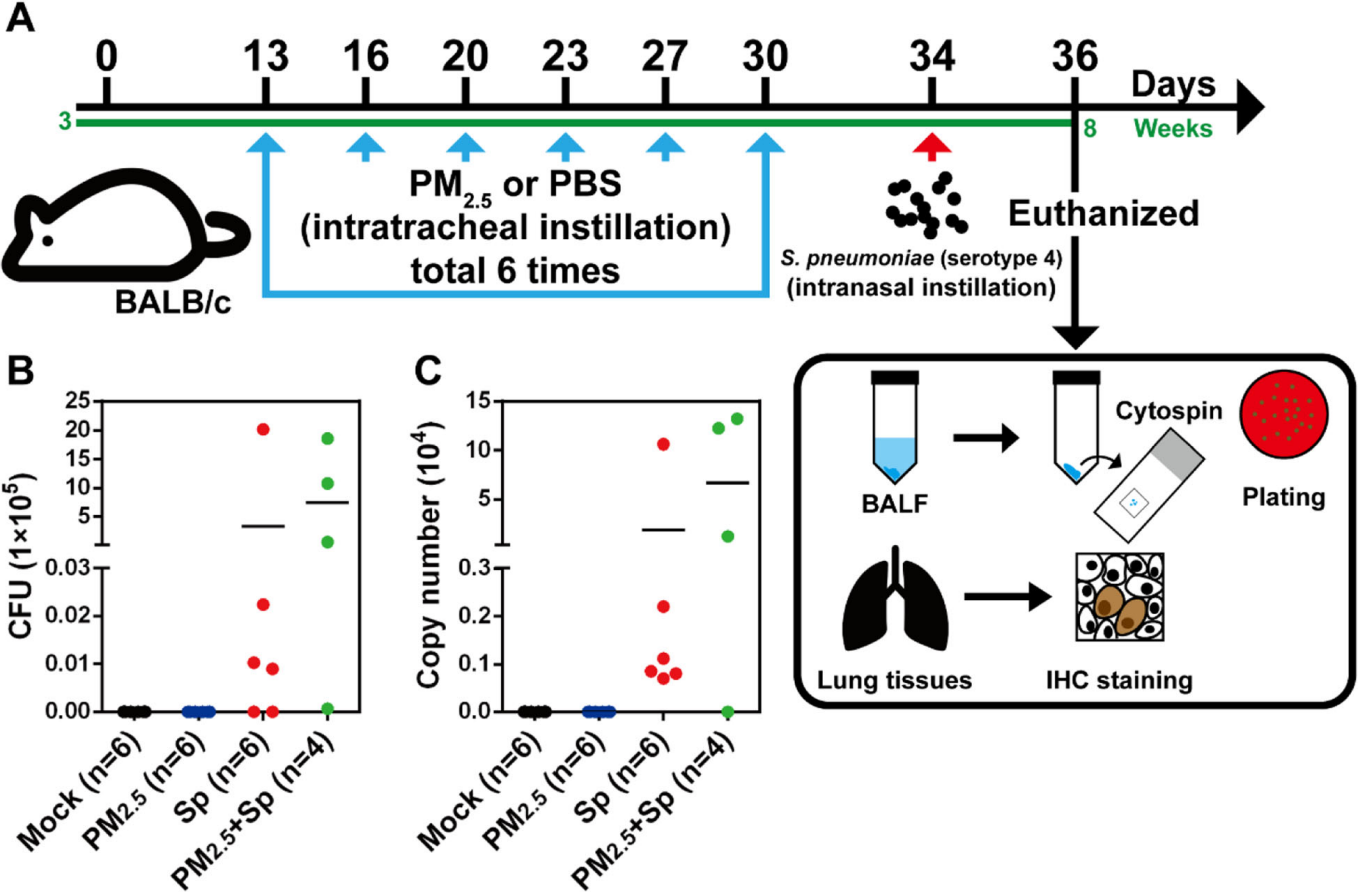
Long-term PM_2.5_ exposure enhances pneumococcal load in the lung. **a** Mice were intratracheally (i.t.) administered PM_2.5_ twice weekly for three weeks (for a total of 200 μg), and were infected with pneumococcus (1 × 10^8^) via intranasal (i.n.) instillation. Mice were euthanized 48 h post-infection, and their BALF harvested for (**b**) bacterial load and (**c**) pneumococcal genomic DNA copy number determination. Horizontal lines indicate the mean value in each treatment group

To ascertain whether long-term PM_2.5_ exposure impairs the immune response and enhances the bacterial burden in host respiratory systems, we established sets of PM_2.5_-exposed murine models (Fig. 4a). Mice were divided into four groups: untreated mock, PM_2.5_-exposed, pneumococcus-infected, and PM_2.5_ + pneumococcus co-treated groups. Mice were exposed to PBS or PM_2.5_, twice weekly, for 3 weeks (for a total of 200 μg), followed by pneumococcal infection for 48 h. The body weights and temperatures of the mice were measured every 3 days, and were not observed to be different among the four groups (Additional file 1: Fig. S2). The mice were then euthanized, BALF was collected, and bacterial loads were determined. Pneumococcal loads and bacterial genomic DNA copy numbers were increased in the PM_2.5_ + pneumococcus co-treated group, compared with those in the only pneumococcus-infected group (Fig. 4b and c). The BALF cells were differentiated using Wright-Giemsa stain. Lymphocyte count was markedly decreased in PM_2.5_ + pneumococcus co-treated mice, compared with those in the other treatment groups (Additional file 1: Table S2). In contrast, eosinophil count was increased in both the PM_2.5_-exposed and PM_2.5_ + pneumococcus co-treated groups.

**Fig. 5:**
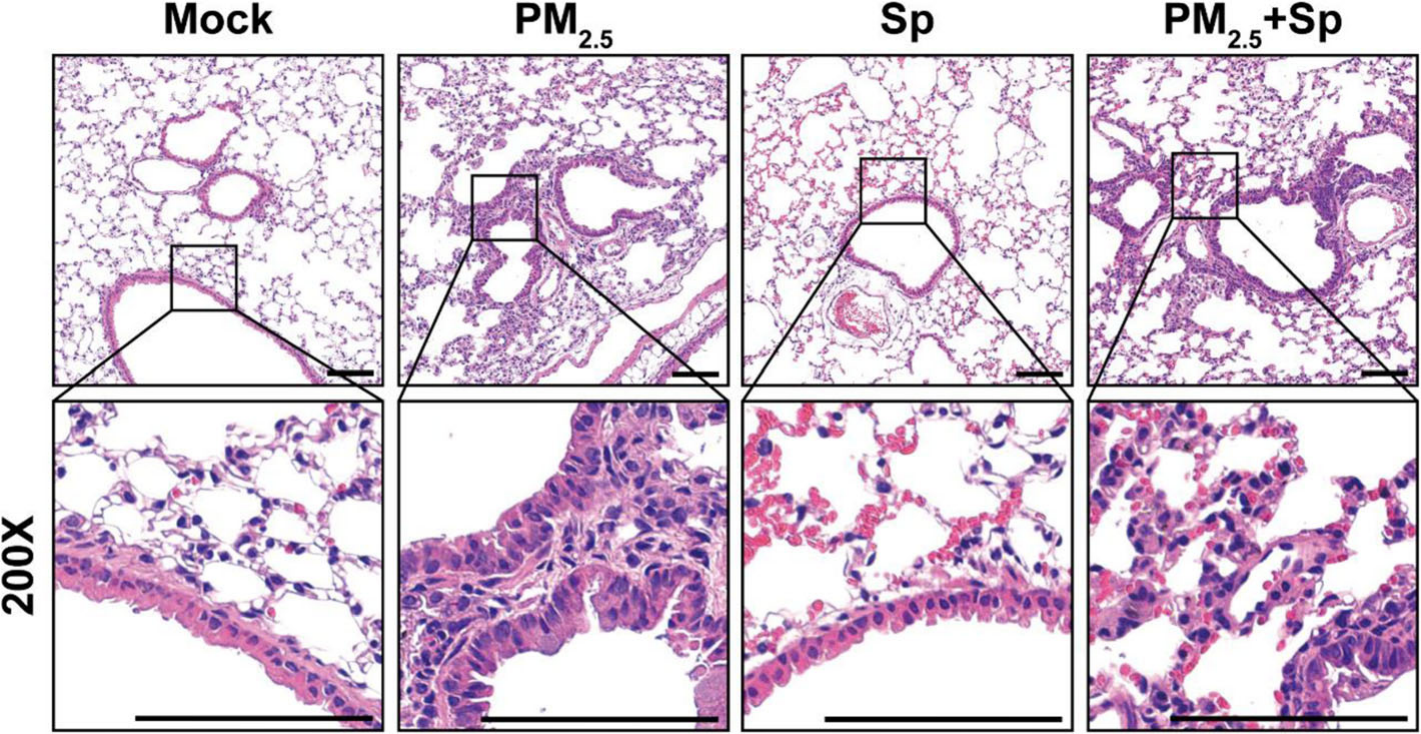
PM_2.5_ aggravates pneumococcus-induced pulmonary pathogenesis**.** Mice were administered PM_2.5_ and pneumococcus-infected as described in Fig. 4. After euthanizing the mice, lung sections were subjected to hematoxylin/eosin (H&E) staining (*n* = 4 per treatment group). The lower panel shows magnified images of the cropped areas. Images were observed under a microscope with 200× magnification. Scale bars, 100 μm

Murine lung tissues were then subjected to H&E and IHC staining. Inflammatory cells were absent around the lung bronchi of untreated and pneumococcus-infected mice (Fig. 5). However, there was notable inflammatory cell infiltration around the bronchi of PM_2.5_-exposed mice. After pneumococcal infection, lung inflammation was noticeable with erythrocyte infiltration in the pulmonary parenchyma. When PM_2.5_-exposed mice were pneumococcus-challenged, immune cell infiltration around the bronchi and erythrocytes in the pulmonary parenchyma were obvious in the lung tissues. Therefore, long-term PM_2.5_ exposure favors bacterial infection of the lungs, which exacerbates inflammation and aggravates pulmonary pathogenesis.

### PM_2.5_ dampens pneumococcus-induced chemokine production

**Fig. 6:**
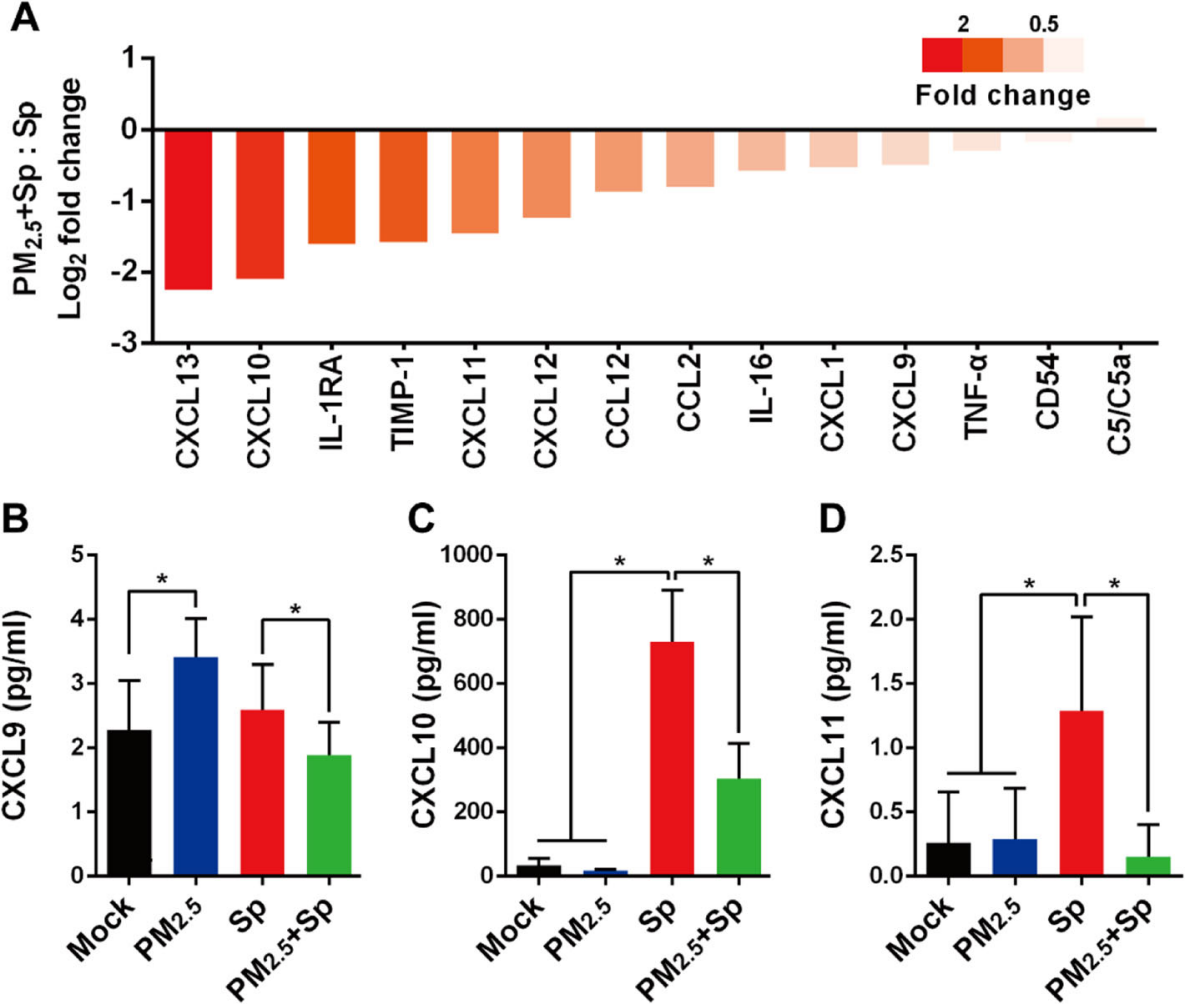
PM_2.5_ inhibits pneumococcus-induced chemokine production. **a** Mice were administered PM_2.5_ and infected with pneumococcus, as described in Fig. 4. After euthanizing the mice, the collected BALF collected from mice (*n* = 6) was pooled into one sample and subjected to cytokine array analysis. Chemokine expression was quantified using ImageJ. Log2 fold changes were calculated for the PM_2.5_ + pneumococcus co-treated and only pneumococcus-infected groups. RAW264.7 cells were incubated with or without 20 μg/ml PM_2.5_ for 24 h, and pneumococcus-infected or uninfected for 6 h. Culture supernatant was collected, and the concentrations of (**b**) CXCL9, (**c**) CXCL10, and (**d**) CXCL11 were determined using ELISA. Statistical significance was analyzed using one-way ANOVA followed by a post-hoc test (*, *P* < 0.05)

To evaluate the effect of PM_2.5_ exposure on chemokine production in mice, chemokines in BALF were analyzed with cytokine array. The expression levels of CXCL13, CXCL10, IL-1RA TIMP-1, CXCL11, CXCL12, CCL12, CCL2, CXCL1, IL-16, CXCL9, TNF-α, and CD54 decreased, whereas that of C5/C5a increased in the PM_2.5_ + pneumococcus co-treated mice compared with those in the pneumococcus-infected mice (Fig. 6a). The ligands, CXCL9, − 10, and − 11, binding to CXCR3 play crucial roles in immune cell activation [34]. We next explored macrophage expression of CXCL9, − 10, and − 11. As expected, CXCL9, − 10, and − 11 secretions were significantly decreased with prior PM_2.5_ exposure, followed by bacterial challenge, compared with that with pneumococcal infection alone (Fig. 6b-d). In addition, IHC analysis showed decreased expression of IL-1β and CXCR3 in the lung tissues of PM_2.5_ + pneumococcus co-treated mice when compared to that in the untreated mice (Additional file 1: Fig. S3). These results suggest that PM_2.5_ exposure attenuates the pneumococcus-induced chemokine production.

**Fig. 7:**
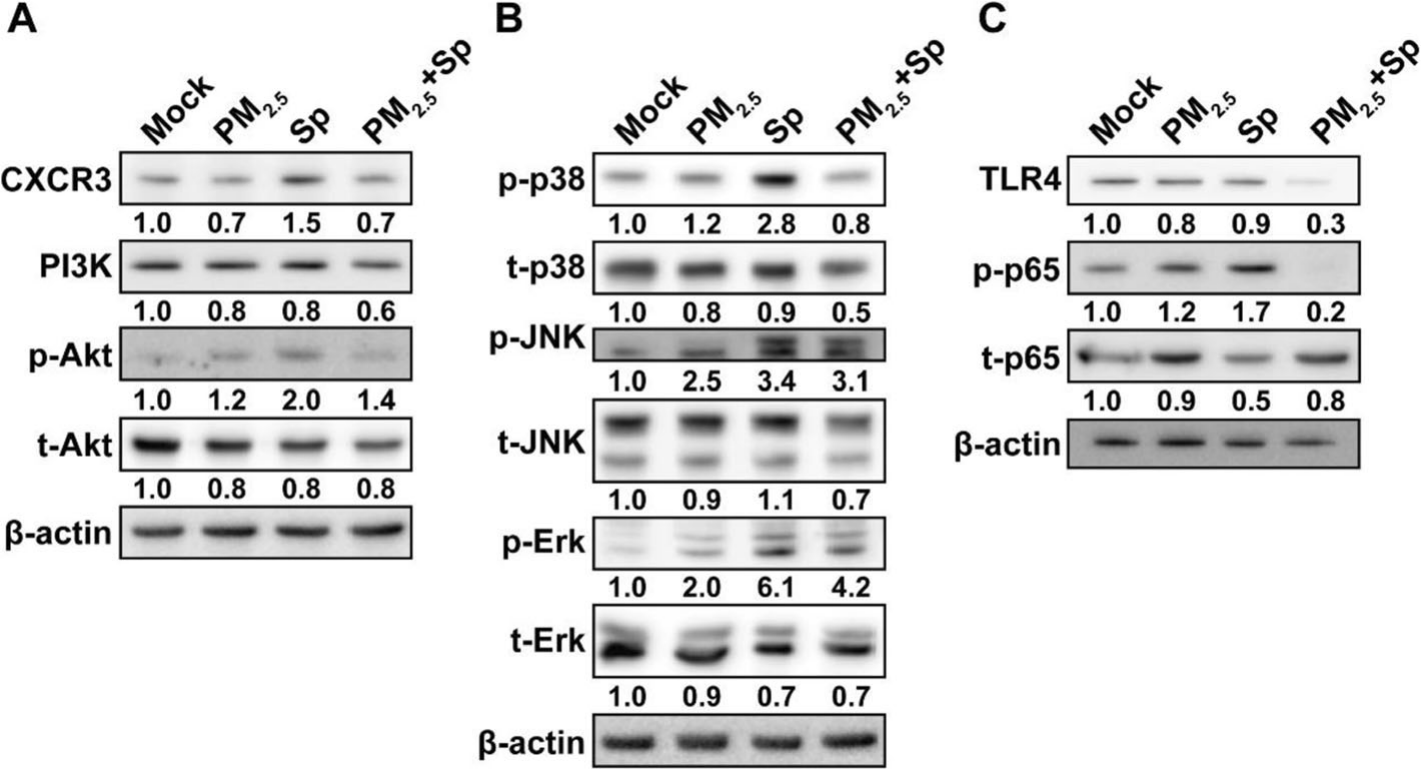
PM_2.5_ impairs pneumococcus-induced chemokine production via the PI3K/Akt and MAPK pathways. RAW264.7 cells were exposed to PM_2.5_ (20 μg/ml) for 24 h, followed by pneumococcus infection for 6 h. Cell lysates were prepared and subjected to western blot analysis using antibodies against (**a**) CXCR3, PI3K, and Akt, (**b**) p38, JNK, Erk, and their respective phosphorylated forms, and (**c**) TLR4, p65, and phosphorylated p65. β-actin was used as a loading control. Relative expression levels were normalized to those in the mock-treated group and are indicated under each band

Because CXCR3-mediated inflammation is regulated by the PI3K/Akt and MAPK pathways [35], we further analyzed the levels of particular molecules involved in CXCR3 signaling. Decreases in CXCL9, − 10, and − 11 levels reduced CXCR3 and PI3K/Akt expression in PM_2.5_ + pneumococcus co-treated macrophages, compared with that for pneumococcal infection alone (Fig. 7a). Furthermore, the phosphorylation of p38, JNK, and Erk was markedly decreased in PM_2.5_ + pneumococcus co-treated cells as opposed to that in pneumococcus-infected cells (Fig. 7b). In parallel, TLR4 expression and p65 phosphorylation noticeably declined in macrophages co-treated with PM_2.5_ + pneumococcus compared with those in the pneumococcus-infected group (Fig. 7c).

**Fig. 8:**
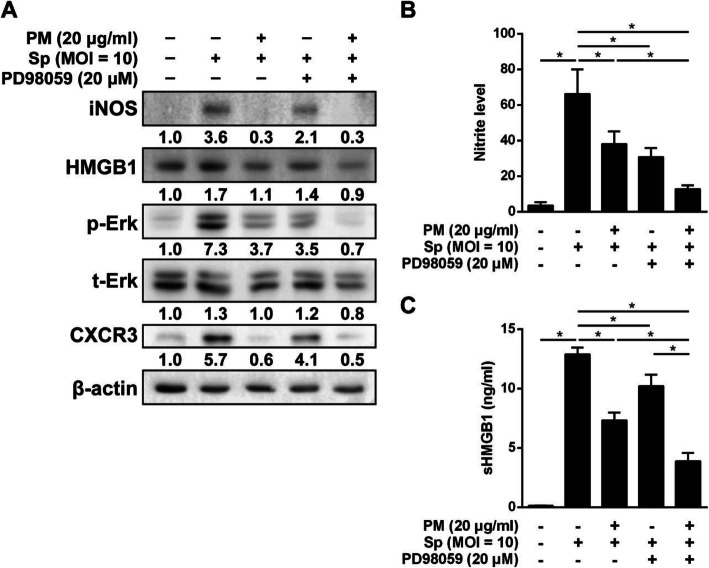
Signaling pathways involved in the inhibition of pneumococcus-induced chemokine production by PM_2.5_**.** RAW264.7 cells were pretreated with 20 μM PD98059 (Erk inhibitor) and exposed to PM_2.5_ (20 μg/ml) for 24 h, followed by pneumococcus infection (MOI = 10) for 6 h. Cell lysates were prepared and analyzed by western blotting using antibodies against (**a**) iNOS, HMGB1, p-Erk, t-Erk, and CXCR3. β-actin was used as a loading control. Relative expression levels were normalized to those in the mock-treated group and are indicated under each band. **b** nitric oxide concentrations were determined using Griess reagent, and (**c**) sHMGB1 production was assessed by ELISA. The data are means ± standard deviations from triplicate independent experiments. Statistical significance was evaluated using one-way ANOVA, followed by a post-hoc test (*, *P* < 0.05)

We then conducted an inhibition assay to analyze the involvement of the MAPK and NF-κB signaling pathways in the macrophage function-impairing effect of PM_2.5_. Macrophages were pretreated with Erk inhibitor (PD98059), which was followed by PM_2.5_ and pneumococcus treatment. As shown in Fig. 8a, PM_2.5_ exposure decreased the level of phosphorylated Erk and the expression of iNOS, HMGB1, and CXCR3 in pneumococcus-infected macrophages. In parallel, pneumococcus-induced iNOS, HMGB1, p-Erk, and CXCR3 were significantly suppressed in cells pretreated with PD98059. Interestingly, the suppressive effect of PM_2.5_ on pneumococcus-induced inflammatory molecules was augmented by PD98059. This trend was also observed during the analysis of NO production and HMGB1 secretion (Fig. 8b-c). Our data further showed that pneumococcus-induced NO production was inhibited by PM_2.5_, SB203580 (p38 inhibitor), or SP600125 (JNK inhibitor) (Additional file 1: Fig. S4). Moreover, our findings suggest that pneumococcus-induced NO production can effectively be suppressed by co-treatment with PM_2.5_ and JSH-23 (NF-κB inhibitor). These results demonstrate that PM_2.5_ inhibits pneumococcus-stimulated macrophage activation through the MAPK and NF-κB signaling pathways.

## Discussion

Air pollution has become a serious public health concern worldwide. PM_2.5_ has a larger surface and can adhere to transient metals, toxic substances, and pathogens, and can be inhaled into the respiratory system [36–38]. A growing number of investigations have indicated that PM_2.5_ exposure is associated with several respiratory diseases, including chronic obstructive pulmonary disease (COPD) and asthma [39, 40]. Noticeably, PM_2.5_ can penetrate the respiratory barrier and enter the circulatory system, therefore spreading throughout the body, leading to cardiovascular diseases, hypertension, diabetes mellitus, and systemic inflammation [2, 41–44]. Alveolar macrophages initially engulf lung-deposited PM_2.5_ [19] that impair the pulmonary immune system, resulting in increased bacterial infectivity [17, 18]. Given the evidence that PM_2.5_ poses adverse health risks, the current study comprehensively explored the mechanism underlying the PM_2.5_-mediated impairment of the primary immune defense against bacterial infection.

Phagocytosis is a strategy by which macrophages trigger lysosomes to degrade internalized bacteria [45]. The activated macrophages then produce proinflammatory cytokines to recruit other immune cells associated with adaptive immunity, to collaboratively eradicate the pathogens [46]. Concentrated ambient particles have been shown to inhibit macrophage bacteria internalization [16]. Oil fly ash exposure damaged the lungs and decreased nitric oxide production, attenuating bacterial clearance by macrophages [47]. Consistent with previous findings, our current results demonstrated that PM_2.5_ subverts macrophage phagocytic activity, and decreases proinflammatory cytokine production in response to pneumococcal infection. Furthermore, the nitric oxide level, which exhibits antimicrobial activity, was suppressed by PM_2.5_-mediated inhibition of iNOS expression. Collectively, these findings provide information on how particulate matter subverts macrophage activity to enhance bacterial infectivity.

High PM_2.5_ exposure levels (≥ 100 μg/ml) are known to enhance proinflammatory cytokine production, resulting in lung toxicity [48–50]. However, in this study, cell viability was unaffected and proinflammatory cytokine production was not significantly increased in macrophages treated with relatively low PM_2.5_ levels (20 μg/ml). In contrast, pneumococcal infection markedly enhanced proinflammatory cytokine secretion; however, this trend was in turn remarkably reduced by PM_2.5_ exposure. The low dose of PM_2.5_ administered to macrophages before pneumococcal challenge may have substantially suppressed proinflammatory cytokine production rather than being cytotoxic. The association between PM_2.5_ exposure at different concentrations and impaired macrophage function following pneumococcal infection merits further investigation.

It has been reported that PM induces inflammatory response through the TLR2 and TLR4 pathways [51, 52]. TLR2 recognizes pneumococcal peptidoglycan and induces an inflammatory response, thus promoting host defense against bacterial infection [53]. In addition, macrophages infected with pneumococcus express a scavenger receptor, macrophage receptor with collagenous structure (MARCO), which is co-expressed with TLR2 and nucleotide-binding oligomerization domain-containing 2 (NOD-2), to regulate inflammatory responses [54]. Further, another macrophage scavenger receptor, SR-AI/II has been reported to be implicated in innate defense against bacteria and TiO_2_ particles [55]. Collectively, these findings indicate that pattern recognition receptors (PRPs) may act as receptors for bacteria and environmental particles. However, very few studies have assessed the synergistic and antagonistic interactions between PM and bacteria. Therefore, the extent to which bacteria and PM activate different, same, or opposing receptors is still unclear and warrants future studies.

HMGB1 is one of the danger-associated molecular pattern (DAMP) proteins, which are endogenous danger signals [56]. HMGB1 is a ubiquitous nuclear protein facilitating NF-κB transcription in eukaryotes [57]. Once HMGB1 is released by necrotic or activated immune cells, it activates the production of extracellular proinflammatory cytokines, including IL-1α, IL-1β, IL-6, IL-8, TNF-α, and IFN-γ [58–60]. Our results showed that HMGB1 production was increased by PM_2.5_-exposure or bacterial infection but decreased by PM_2.5_ + pneumococcus co-treatment. A similar trend was also observed for the expression level of phosphorylated p65. PM_2.5_-treatment plus bacterial infection inhibited the production of proinflammatory cytokines including IL-1α, IL-1β, and TNF-α, thereby enhancing bacterial infectivity. HMGB1 appears to significantly manipulate macrophage proinflammatory cytokine production during bacterial infection. However, the mechanism underlying PM_2.5_ suppression of HMGB1-mediated immune defense against pneumococcus remains to be elucidated.

Macrophages can be polarized into two phenotypes: M1 (proinflammatory phenotype) and M2 (anti-inflammatory phenotype) [61]. Previous studies indicated that PM_2.5_ exposure significantly induced the inflammatory M1 polarization, which contributed to lung disorders [62, 63]. In contrast, recent investigations demonstrated that PM_2.5_ activated M2-polarization to exacerbate lung eosinophilia and allergic responses [64, 65]. However, discrepancies and controversial results have emerged. We, therefor, conducted additional studies to assess the effects of PM_2.5_ on macrophage phenotypic transition. The obtained ELISA and qRT-PCR results demonstrated that pneumococcal infection activated M1 macrophages (Fig. 3f-g and Additional file 1: Fig. S5A-C), which is in accordance with the results of previous studies [66, 67]. Noticeably, PM_2.5_ suppressed pneumococcus-induced M1 macrophage markers. In addition, IHC analysis showed that F4/80^+^ cells were increased in mice infected with pneumococcus when compared with mice in mock-control group (Additional file 1: Fig. S5F-G). Therefore, these findings indicate that PM_2.5_ manipulates macrophage polarization that is possibly responsible for the observed macrophage dysfunction, and it may impair the elimination of bacterial infection, thereby exacerbating inflammation.

**Fig. 9:**
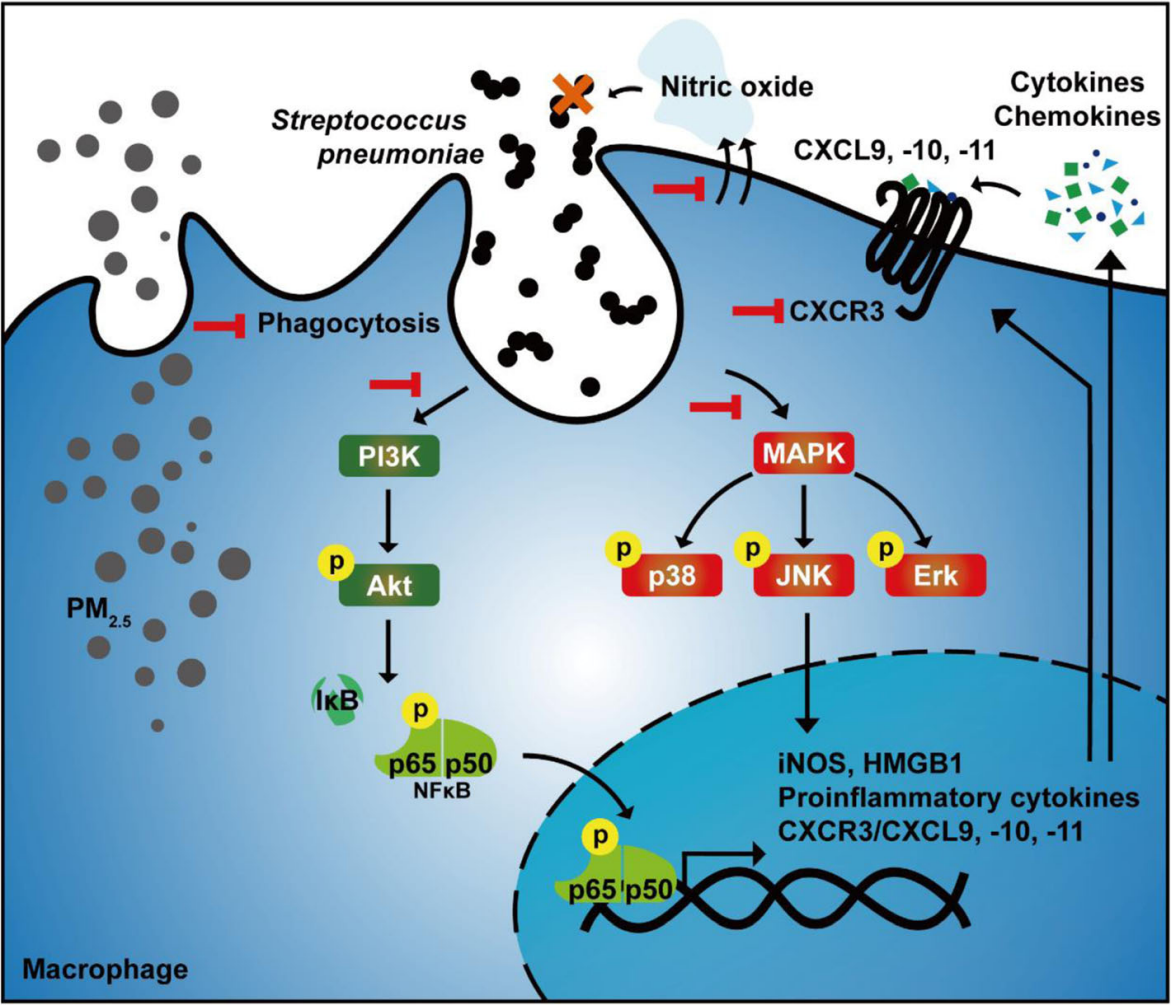
Schematic model illustrating the mechanism underlying the PM_2.5_-mediated dampening of pneumococcus-induced macrophage activation and pulmonary infection exacerbation**.** PM_2.5_ exposure suppresses macrophage phagocytic activity and nitric oxide production during pneumococcal infection. Further, PM_2.5_ subverted proinflammatory cytokines and chemokines by inhibiting the PI3K/Akt and MAPK pathways in pneumococcus-infected macrophages

The CXCR3 ligands, including CXCL9, − 10, and − 11, generate Th1 response [34]. CXCL10/CXCR3 interaction is essential for promoting immune cell functions, including differentiation, migration, and activation [34]. Activation of the macrophage CXCL10/CXCR3 axis is regulated by the PI3K/Akt and MAPK pathways [68]. Additionally, CXCL10 production is positively associated with pneumococcal load [69]. Our results expanded the prior findings and showed that CXCL9, − 10, and − 11 expression in BALF was significantly increased after pneumococcal infection, but decreased upon PM_2.5_ treatment. Furthermore, we identified that pneumococcus-induced CXCL10/CXCR3, MAPK, and NF-κB signaling pathway activation was suppressed by PM_2.5_ (Fig. 9). Impaired CXCL10 expression increases the susceptibility to bacterial infection [70]. These findings support our results that PM_2.5_ decreases CXCL10/CXCR3 activation, thus contributing to pneumococcus-induced pathogenesis.

Pneumococcus is one of the most crucial human pathogens causing community-acquired pneumonia [71]. The serotypes 1, 4, 6B, 7F, 14, and 19F of pneumococcal isolates are known to have an invasive disease potential in humans [72]. The pneumococcal strain, serotype 4 (TIGR4) causes the most-severe invasive disease, while the serotype 1 clones cause low-level bacteremia without any disease symptoms [73]. In addition, the completely sequenced genome of the TIGR4 strain has been extensively employed in laboratory animal models to elucidate the pathological features of pneumococcal pneumonia and sepsis [74]. Therefore, due to its high virulence and poor pathogenic outcomes, we chose the TIGR4 strain for this study.

Although the mechanisms underlying the PM_2.5_-mediated dampening of macrophage activity against pneumococcal infection were investigated in this study, the cell-based and animal studies may not completely reflect the pathophysiology in humans. Moreover, it is difficult to collect BALF from PM_2.5_-exposed pneumococcal-infected patients. Further investigations should analyze samples from pneumococcus-infected patients living in PM_2.5_-polluted urban areas to fill the gap for the translational utility of the present study.

## Conclusions

In the present study, we provided evidence that PM_2.5_ exposure impairs macrophage functions, including phagocytosis and nitric oxide production, which, in turn, hampers bacterial clearance activity. Additionally, PM_2.5_ perturbs the macrophage polarization and may cause macrophage dysfunction. Animal studies showed that long-term PM_2.5_-exposure inhibits pneumococcus-induced production of proinflammatory cytokines, favors pneumococcal infection, and exacerbates pulmonary pathogenesis. We further demonstrated that PM_2.5_ exposure dampens pneumococcus-induced chemokine and CXCR3 production through suppression of the PI3K/Akt and MAPK signaling pathways. Our findings provide novel translational insight into the mechanism underlying PM_2.5_-induced aggravation of pneumococcal infection of the airways.

## Supplementary information

**Additional file 1: Figure S1.** Deposition of particulate matter in the cytoplasm. RAW264.7 cells were (A) untreated (mock) or (B) treated with 20 μg/ml PM_2.5_ for 24 h and subjected to light microscopic analysis. Arrows represent macrophages with phagocytosed PM_2.5_. Scale bars, 5 μm. **Figure S2.** The body weight and temperature of mice during the experiment. Mice were divided into four groups (10 per group) for the treatments with PBS (mock), PM_2.5_, pneumococcus (Sp), and PM_2.5_ + pneumococcus, respectively. The body weights and temperatures of the mice were measured every three days during the experiment. **Figure S3.** Long-term PM_2.5_-exposure decreases the expression of CXCR3 and IL-1β in pneumococcus-infected lung tissues. Mice were administered PM_2.5_ and pneumococcus-infected as described in Fig. 4. Mice were euthanized and lung tissues were subjected to immunohistochemical (IHC) staining with specific antibody against CXCR3 and IL-1β (original magnification: 200×). Magnified images of each cropped area are shown in the lower panel. Scale bars, 100 μm. **Figure S4.** Involvement of the MAPK and NF-κB signaling pathways in the suppression of pneumococcus-induced nitric oxide production by PM_2.5_. RAW264.7 cells were pretreated with SB203580 (10 μM), SP000125 (10 μM), or JSH-23 (20 μM) for 1 h, and exposed to PM_2.5_ (20 μg/ml) for 24 h, followed by pneumococcal infection for 6 h. The nitric oxide concentration was determined using Griess reagent. Statistical significance was evaluated using one-way ANOVA followed by a post-hoc test (*, *P* < 0.05) to compare with pneumococcus infection alone group. **Figure S5.** PM_2.5_ subverts the macrophage polarization**.** RAW264.7 cells were unexposed or exposed to 20 μg/ml of PM_2.5_ for 24 h, followed by pneumococcal challenge for 6 h. mRNA levels of (A) iNOS, (B) CD80, (C) CD86, (D) CD163, (E) CD206, and (F) F4/80 in macrophages were determined by qRT-PCR analysis. Data are presented as means ± standard deviations from triplicate independent experiments. Statistical significance was analyzed using one-way ANOVA, followed by a post-hoc test (*, *P* < 0.05). (G) Mouse lung tissues were subjected to immunohistochemical (IHC) and stained with anti-F4/80 antibody (original magnification: 200×). Magnified images of each cropped area are shown in the lower panel. Arrows in red indicated F4/80-positive cells. Scale bars, 100 μm. **Table S1.** Primers used for qRT-PCR. **Table S2.** Differentiated cell counts in BALF.

## Data Availability

All the data and materials presented in the current study along with additional files are available.

## Abbreviations

AMP: Antimicrobial peptide
CFA: Coal fly ash
COPD: Chronic obstructive pulmonary disease
CFU: Colony-forming unit
ELISA: Enzyme-linked immunosorbent assay
FE-SEM: Field-emission scanning electron microscope
FITC: Fluorescein isothiocyanate
HMGB1: High mobility group box 1 protein
IFNγ: Interferon-γ
IL-1: Interleukin-1
iNOS: inducible nitric oxide synthase
MOI: Multiplicity of infection
NO: Nitric oxide
NF-κB: Nuclear factor kappa B
PM_2.5_: Ambient fine particulate matter
ROS: Reactive oxygen species
SDS: Sodium dodecyl sulfate
TNF: Tumor necrosis factor
