# Supplementary figures and images for: PM2.5 impairs macrophage functions to exacerbate pneumococcus-induced pulmonary pathogenesis
Source: Part Fibre Toxicol. 2020 Aug 4;17:37. doi: 10.1186/s12989-020-00362-2 (PMC7409448; doi:10.1186/s12989-020-00362-2)

Fig. S1

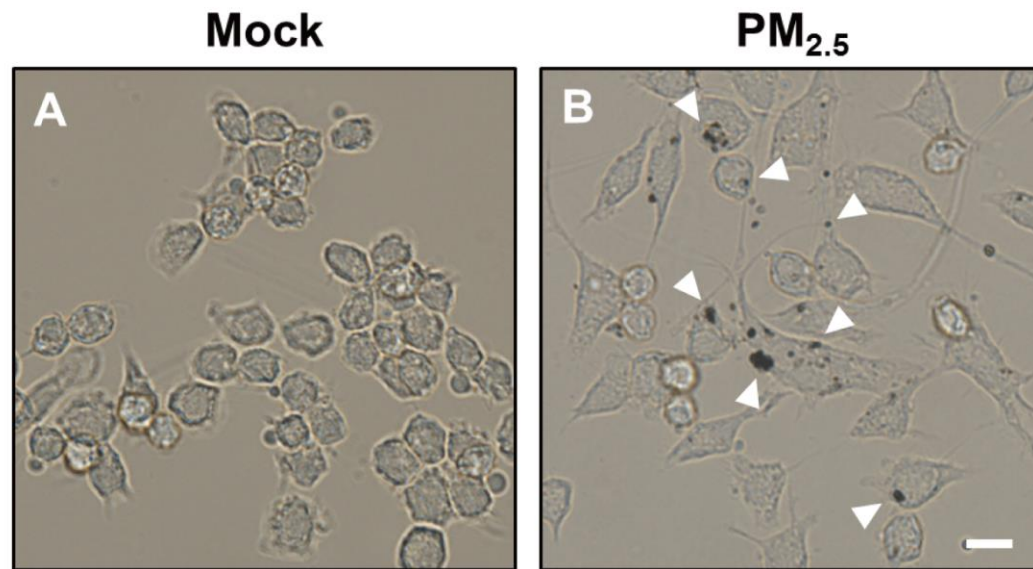

Fig. S2

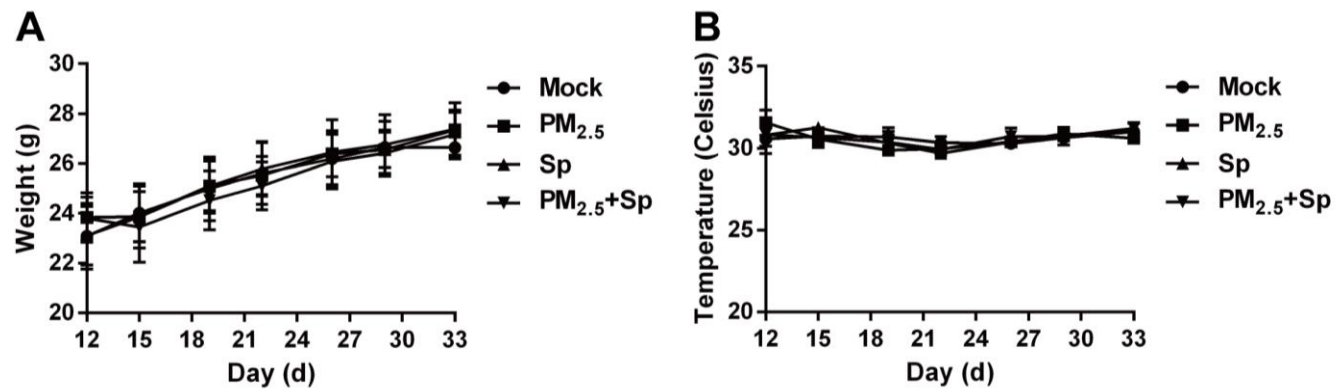

Fig. S3

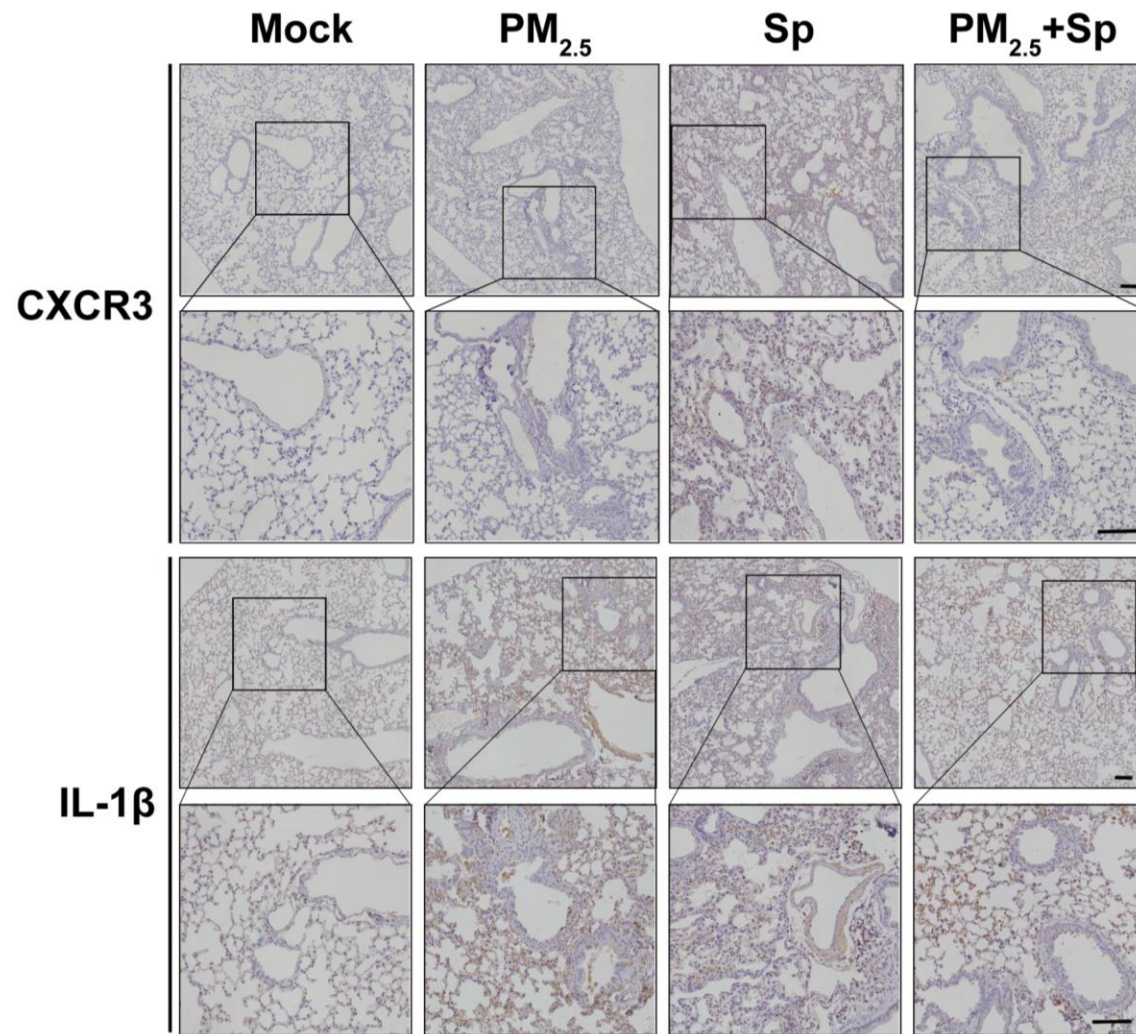

Fig. S4

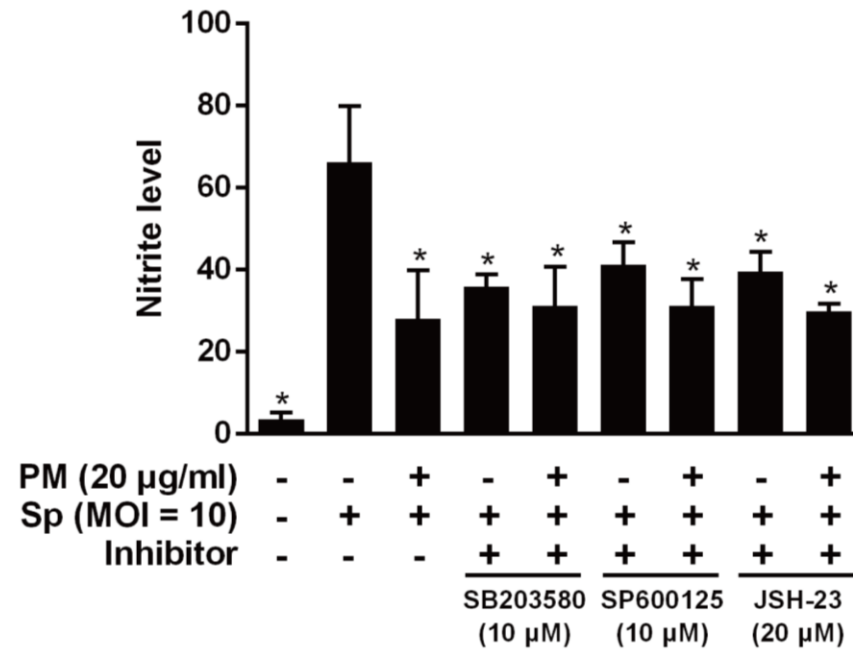

Fig. S5

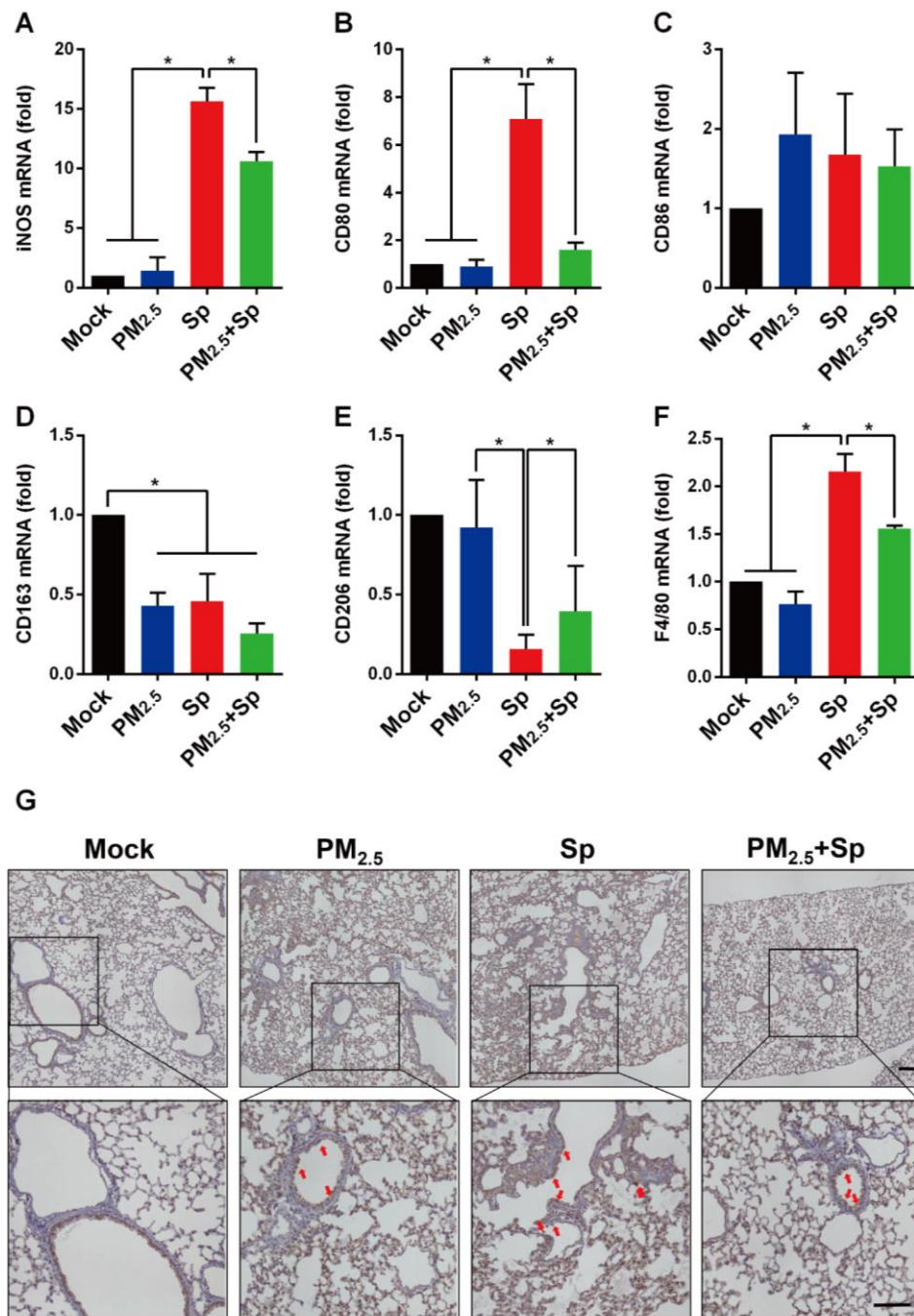

Supplement: Supplementary file 1 — Additional file 1: Figure S1. Deposition of particulate matter in the cytoplasm. RAW264.7 cells were (A) untreated (mock) or (B) treated with 20 μg/ml PM2.5 for 24 h and subjected to light microscopic analysis. Arrows represent macrophages with phagocytosed PM2.5. Scale bars, 5 μm. Figure S2. The body weight and temperature of mice during the experiment. Mice were divided into four groups (10 per group) for the treatments with PBS (mock), PM2.5, pneumococcus (Sp), and PM2.5 + pneumococcus, respectively. The body weights and temperatures of the mice were measured every three days during the experiment. Figure S3. Long-term PM2.5-exposure decreases the expression of CXCR3 and IL-1β in pneumococcus-infected lung tissues. Mice were administered PM2.5 and pneumococcus-infected as described in Fig. 4. Mice were euthanized and lung tissues were subjected to immunohistochemical (IHC) staining with specific antibody against CXCR3 and IL-1β (original magnification: 200×). Magnified images of each cropped area are shown in the lower panel. Scale bars, 100 μm. Figure S4. Involvement of the MAPK and NF-κB signaling pathways in the suppression of pneumococcus-induced nitric oxide production by PM2.5. RAW264.7 cells were pretreated with SB203580 (10 μM), SP000125 (10 μM), or JSH-23 (20 μM) for 1 h, and exposed to PM2.5 (20 μg/ml) for 24 h, followed by pneumococcal infection for 6 h. The nitric oxide concentration was determined using Griess reagent. Statistical significance was evaluated using one-way ANOVA followed by a post-hoc test (*, P < 0.05) to compare with pneumococcus infection alone group. Figure S5. PM2.5 subverts the macrophage polarization. RAW264.7 cells were unexposed or exposed to 20 μg/ml of PM2.5 for 24 h, followed by pneumococcal challenge for 6 h. mRNA levels of (A) iNOS, (B) CD80, (C) CD86, (D) CD163, (E) CD206, and (F) F4/80 in macrophages were determined by qRT-PCR analysis. Data are presented as means ± standard deviations from triplicate independent experi [file 12989_2020_362_MOESM1_ESM.zip › Additional file 1_R2.pdf]
